# Obesity, overweight and breast cancer: new clinical data and implications for practice

**DOI:** 10.3389/fonc.2025.1579876

**Published:** 2025-03-27

**Authors:** Laura García-Estévez, Marta González-Rodríguez, Isabel Calvo, Alberto Orta, María Gión, Gema Moreno-Bueno, José Manuel Pérez-García, Javier Cortés

**Affiliations:** ^1^ Breast Cancer Department, MD Anderson Cancer Center, Madrid, Spain; ^2^ Centro de Investigación Biomédica en Red de Cáncer (CIBERONC), Instituto de Salud Carlos III, Madrid, Spain; ^3^ Department of Medical Oncology, Ramón y Cajal University Hospital, Madrid, Spain; ^4^ Biochemistry Department, Universidad Autonoma de Madrid (UAM), Instituto de Investigaciones Biomedicas ‘Alberto Sols’ (CSIC-UAM), Madrid, Spain; ^5^ MD Anderson Cancer Center Foundation, Madrid, Spain; ^6^ International Breast Cancer Center (IBCC), Pangaea Oncology, Quiron salud Group, Barcelona, Spain; ^7^ Medica Scientia Innovation Research (MEDSIR) - Oncoclínicas&Co, Jersey City (New Jersey, USA), Sao Paulo, Brazil; ^8^ Universidad Europea de Madrid, Faculty of Biomedical and Health Sciences, Department of Medicine, Madrid, Spain

**Keywords:** leptin, breast cancer, bodyweight, obesity, microenvironment

## Abstract

Excess bodyweight has negative consequences in breast cancer (BC) patients, significantly increasing the incidence of BC and adversely affecting clinical outcomes in most BC subtypes. This article overviews recent evidence relating to excess bodyweight (particularly obesity) and its effect on treatment in women with BC, focusing on latest evidence, including clinical findings from recently introduced new therapeutic entities. There is evidence of an inverse relationship between obesity and BC in premenopausal women highlighting a complex interplay involving the tumor microenvironment and tumor cells, and patient factors such as hormonal/metabolic/inflammatory status. Advancements in targeted- and immune-therapy have brought renewed optimism for women with BC. Ultimately, a better understanding of the mechanistic link between adipogenicity and tumorigenicity in breast tissues, as well as how obesity and adipose tissue inflammation interact with female sex hormones, may prove to be an important area for further refinements in our quest to develop a truly personalized therapeutic approach in this clinical setting.

## 1 Introduction

Obesity represents an escalating pandemic which is associated with an increased risk of various cancer types, including breast cancer (BC) (1, 2). Additionally, it elevates the likelihood of developing a number of chronic conditions such as metabolic syndrome, diabetes and heart disease (2). The negative impact of obesity in BC patients is two-fold; firstly, it is associated with a significant increase in BC incidence and, secondly, it is also linked to worse treatment outcomes in most subtypes of the disease (3). However, an exception to this trend is observed in premenopausal women, among whom there is evidence of an inverse relationship between obesity and BC (4). This underscores the intricate interplay involving the tumor microenvironment (TME) and patient factors such as menopausal/hormonal status, metabolic status/lipid metabolism (e.g. hyperlipidemia and hyperinsulinemia), adiposity and inflammatory status (5). Adipose tissue releases inflammatory mediators such as tumor necrosis factor alpha (TNFα) and interleukin (IL)-6 while reducing the production of adiponectin. This predisposes to a pro-inflammatory state characterized by increased oxidative stress and is proposed to be a key player in the initiation and progression of BC in obese people. As such, BC patients with obesity present many unique challenges during cancer treatment but, to date, there are few specific recommendations tailored to meet the needs of obese patients.

In this overview, we performed a literature search of PubMed without date restrictions to investigate the most recent evidence from the published literature, including findings from our own institution, to gain a better understanding of the influence of excess bodyweight on treatment outcomes in patients with BC. While assessment of clinical outcomes is very important, especially with respect to new therapeutic approaches, we also hoped to gain more information in relation to the intricate interplay between adipocyte-associated factors and BC progression. A better understanding of the different aspects of BC that can be adversely affected by obesity may help elucidate potential avenues for new and novel therapeutic interventions.

## 2 Worldwide obesity facts

The World Health Organization (WHO) and other organizations such as the American Medical Association now regard obesity as a chronic disease and a major pandemic in the 21st century (1, 6). In 2022 the WHO estimated that 2.5 billion adults (≥18 years) in the world were overweight and 890 million of these are considered to be obese (1). More recently it has been estimated that global levels of overweight and obesity (BMI ≥25 kg/m²), will exceed 4 billion people by 2035, compared with over 2.6 billion in 2020 (7). This is more than a 3-fold increase since 1975. Although BMI is not considered an accurate tool for determining excess adipose tissue, it is currently the most practical and reliable way to measure it in clinical practice. In Western nations including Europe and the USA, a BMI of 25 kg/m^2^ or more is generally considered to be overweight and 30 kg/m^2^ or more to be obese. In contrast, in Asian countries (8), overweight is usually considered to be ≥23 kg/m^2^ and obesity ≥25 kg/m^2^. This change in BMI range between Western and Asian countries is important when making comparisons or when analyzing the BMI status of patients included in international clinical trials.

In most European countries and the USA excessive bodyweight is a real concern and what is really dramatic is the forecast of global prevalence rates for the year 2030: the proportion of the adult population considered to be overweight or obese being almost 60% (9). This situation will also lead to skyrocketing numbers of patients with diseases linked to excess adipose tissue such as metabolic disorders, cardiovascular diseases, kidney diseases, diabetes, mental illness, cancer and others.

## 3 Obesity and breast cancer: the role of adipokines

Obesity is linked to the development of numerous different types of cancer including BC in postmenopausal women. In contrast, obesity appears to be a protective factor in the development of premenopausal BC. Considering the limitations of BMI to capture the whole excess of adipose tissue, there is currently a lack of data regarding differences in fat mass distribution between pre- and postmenopausal women and the risk for BC. Fat mass distribution is quite different according to menopausal status (10). It remains unknown whether premenopausal women have a different risk for BC than postmenopausal women due to differences in body fat distribution (arms, legs, and trunk). The largest and most recent analysis assessing the association between obesity and BC was undertaken by The Premenopausal Breast Cancer Collaborative Group (4). This multicenter study pooled individual-level data from 758,592 premenopausal women (aged 18-54 years) included in 19 prospective cohorts to estimate hazard ratios (HRs) of premenopausal BC in association with BMI. With a median follow-up of 9.3 years, it was estimated that there was a 12% to 23% reduction in premenopausal BC risk per-5.0-U difference in BMI depending on age. The explanation for this negative association is still unknown and many factors may be involved such as blood estrogen levels and anovulatory cycles. Also, the levels of adipokines, leptin and adiponectin, released by adipocytes and insulin levels could play a key role in this situation (11).

With respect to physiological/metabolic effects, adiponectin and leptin are among the most relevant adipokines released by adipose tissue (5). While leptin has been considered one of the main drivers connecting obesity and BC through activation of the JAK2/STAT3 pathway, adiponectin might have a more protective effect against BC via its effects on the AMP-activated protein kinase (AMPK) signaling pathway (5). Zhao and colleagues conducted an elegant study to evaluate the differential impact of obesity in the breast according to menopausal status (12). Random fine needle aspirates (rFNAs) of breast tissue were collected from 57 premenopausal and 55 postmenopausal healthy women (aged 35-60) classified as normal-weight, overweight, and obese. The samples were taken within 14 weeks of having a normal mammogram. Expression levels of 21 target genes were determined using a TaqMan Low Density Array procedure. Interestingly, *RPS6KB1*, an AMPK downstream-responsive gene for protein synthesis and cell growth, estrogen receptor α (encoded by the *ESR1* gene) and its target gene *GATA3*, were significantly decreased in rFNAs of premenopausal, obese women. In contrast, in postmenopausal women, the expression of these genes remained unchanged in relation to adiposity. These results shed some light into the different potential mechanisms underlying the effects of obesity in the breast depending on menopausal status.

The most recent meta-analysis investigating the role of adipokines levels and BC risk reported that high-risk factors for BC included elevated leptin and lower adiponectin levels, and this was especially the case in postmenopausal women (13). This was highlighted when a combined analysis involving pre- and postmenopausal women was performed which clearly showed a trend linking elevated adiponectin levels with reduced BC risk. These results appear to be driven by a stronger association in the postmenopausal group but, unfortunately, similar studies investigating an association between adiponectin levels and BMI in BC are lacking.

Based on Zhao´s study (12), we conducted, for the first time, a cross-sectional study in premenopausal women with a BMI equal to or greater than 25 kg/m^2^, subdividing them into two cohorts: cohort 1 included a recent diagnosis of early BC and patients in cohort 2 were cancer free (14). Our hypothesis focused on whether the protective role of obesity in premenopausal stages might be in part due to high adiponectin levels and its action through AMPK signaling pathway activation according to Zhao results (12). Thus, we compared leptin and adiponectin blood levels between the two cohorts as well as other potential markers such as insulin and vitamin D levels (14). Of the 86 women included in the analysis, 54 were BC-free and 32 had BC (luminal subtype n=23, triple negative n=3 and human epidermal growth factor 2 [HER2]-positive [HER2+] n=6). With regard to our hypothesis, there were no differences in adiponectin levels either as a continuous variable or in the categorization between BC and BC-free women. Furthermore, women without BC had significantly higher BMI (median 32.3 vs 26.8 kg/m^2^; p<0.001), leptin levels (median 27.3 vs 12.8; p<0.001), and insulin levels (median 10.8 vs 6.8; p<0.003) than BC patients. Leptin values were not significantly different in the various BC subtypes. However, a notable finding was a significant correlation between high leptin and insulin levels which was only observed in BC patients with obesity (p<0.001). This correlation is not observed in overweight patients with cancer or non-cancer patients. Although our findings do not hold true for adiponectin levels, what is interesting is that, although leptin is tumorigenic and high leptin levels are associated with BC, patients without cancer had higher leptin levels than cancer patients. The correlation between leptin and insulin levels was only significant in cancer patients with obesity raising the question of the potential role of the insulin pathway and insulin-like growth factor-1 (IGF-1) as a mitogenic factor in tumorigenesis.

Excess adipose tissue, in addition to favoring a state of chronic inflammation in the microenvironment, produces a situation of insulin resistance and, therefore, high levels of insulin in the blood as well as other factors related to the insulin pathway such as IGF-1. In fact, circulating plasma insulin/IGF-1 concentrations have been shown to independently predict the risk of certain tumor types in humans (15). However, regarding the presence of type 2 diabetes and insulin levels and the risk of developing BC, the results are contradictory according to the menopausal status of the woman and are independent of BMI. For example, a meta-analysis of 20 studies from nine different countries showed a 20% increase in BC risk, independent of BMI, in postmenopausal women with diabetes compared with individuals who were not diabetic; significance was lost in premenopausal women (16).

## 4 Breast tumor microenvironment in obese and non-obese patients

BC progression does not only depend on the tumor subtype, or on genetic changes in the tumor; the interaction with the cellular microenvironment surrounding the tumor is also crucial, not only for tumor growth but also for tumor progression and resistance to therapies.

The TME is key in promoting tumor growth, progression and resistance to specific treatments. Recent data report that the TME may differ according to BC subtype, leading to the hypothesis that response to treatment may also be influenced by the microenvironment (17). The microenvironment component can be classified as local or regional and includes the extracellular matrix (ECM). The local component includes the tumor cells themselves as well as intratumoral cells corresponding to immune cells such as lymphocytes, plasma cells, macrophages, dendritic cells, and neutrophils. Tumor-infiltrating lymphocytes (TILs) constitute a relevant population in certain BC subtypes, mainly triple negative and HER2+ tumors (17). They also have prognostic and predictive value in early BC. The majority of TILS are CD8 cytotoxic T-cells that eliminate the tumor and to a lesser extent CD4 T-cells. Regulatory T-cells (Treg) are important for maintaining immune system homeostasis and tolerance, and their presence in the TME promotes immunosuppression through immunosuppressive cytokines and direct cell-cell interaction. Tumor-associated macrophages (TAMs) are the most frequent innate immune cells in tumors and can present two different phenotypes (M1 proinflammatory or M2 anti-inflammatory) depending on the expression of cytokines released by T cells (17).

Regarding the regional component, this refers to the area of stroma adjacent to the tumor cells. In this component, the most abundant cells are cancer-associated fibroblasts (CAF). These cells can induce tumor progression by promoting angiogenesis, tumor growth and invasion (18). CAF are a heterogeneous group of cells that are morphologically fibroblast-like. They are not necessarily derived from transformation of normal fibroblasts in the TME, since they may also originate from different tissues or precursor cells. The presence of a fibrotic focus within the tumor is now considered a poor prognostic factor (19). This fibrotic focus may correspond to fibroblast cell proliferation or marked hyalinization. Myoepithelial cells, adipocytes, and endothelial and vascular/lymphatic endothelial cells are other cells that make up the stroma. There is an interaction between these constituents and tumor cells in such a way that BC cells stimulate angiogenesis via endothelial growth factor (EGF). Adipocytes are the major cellular components of adipose tissue and play an important role in maintaining energy balance. For many years, adipocytes were considered primarily as cells that stored fat, but now it is known that there are more than 600 proteins released by adipose tissue, and adipocytes are responsible for the production and release of numerous adipokines (20).

The largest component of the TME is the ECM, which is composed of proteins such as collagen, proteoglycans, hyaluronic acid, and laminins (21). The ECM is crucial for the maintenance of the TME and the induction of metastasis. Aside from acting as a physical cellular scaffold, it is responsible for cellular adhesion and migration out of the TME, and regulates cell proliferation, differentiation, and survival (21).

In the case of overweight and obesity in patients with BC, the TME reflects a state of chronic inflammation that further promotes cancer progression. Obesity can materially alter adipose tissue histology and function as a result of adipose tissue expansion stemming from increased energy-storage demands (22). As adipocytes increase in size (hypertrophize), some become apoptotic and are surrounded by macrophages to form crown-like structures that have become a hallmark of adipose tissue inflammation (22). With progressive weight increase and the development of obesity, leptin levels increase and the release of large amounts of leptin by adipocytes helps maintain a pro-inflammatory microenvironment (23). Leptin exerts its function through binding to leptin receptor (Ob-R). This receptor is hardly expressed in normal glandular epithelium, but is highly expressed in BC cells. In a study conducted by our group, Ob-R was significantly expressed in patients with high BMI as well as in triple-negative and HER2+ BC subtypes and, interestingly, was predictive of response to neoadjuvant chemotherapy treatment (24). Obesity-induced leptin expression, in combination with major histocompatibility complex class II (MHCII) expression (by adipocytes and other cell types) stimulates T-helper type 1 (TH1) cell activation. Coupled with this, natural killer, CD8+ T, and activated TH1 cells secrete interferon-γ which, in turn, stimulates adipocyte MHCII expression and M1 macrophage polarization, and may also reduce Treg function as adipose expansion progresses (23, 24). Pro-inflammatory cytokines, including IL-1β, IL-6, and TNFα, that exacerbate adipose inflammation, are produced by adipocytes and M1-like macrophages. These processes are paralleled by a decrease in Treg and TH2 cells which are known anti-inflammatory cells (23, 24).

The extent to which adipose tissue influences the tumor microenvironment and, specifically, the immune system is illustrated in the study by Floris et al. (25). In this retrospective study of over 400 patients with early triple-negative BC (TNBC) treated with neoadjuvant chemotherapy, the impact of adipose tissue on TILs and pathological complete response (pCR) was evaluated. BMI modified the effect of stromal TILs on pCR and prognosis in TNBC patients. Indeed, when defining highly infiltrated tumors as those with at least 30.0% TILs, a pCR rate of 73.1% was recorded in lean patients compared with 44.7% in overweight/obese patients. It is clear that in some way adipose tissue modulates the action of lymphocytes, although the authors were unable to provide a clear explanation. In this regard, we have performed a similar study in 87 patients with early HER2+ BC treated with neoadjuvant chemotherapy and anti-HER2 treatment; 27 of 85 patients were overweight/obese (BMI ≥25 kg/m^2^) (26). Ob-R overexpression was significantly correlated with a BMI ≥ 25 kg/m^2^ as we demonstrated in our previous study. Tumors with Ob-R overexpression had significantly higher levels of stromal TILs than those with non-overexpressed Ob-R (23% vs 16% p=0.007). Despite having higher levels of TILs, the rate of pCR in patients with overexpressed Ob-R was not higher than in patients with Ob-R which was not over-expressed (63% vs 59%; p=0.704). This could be due to the fact that Ob-R overexpressed tumors which would correspond to patients with a high BMI had significantly higher PD-1 expression than Ob-R negative tumors (median 4 vs 1; p=0.012). PD-1 expression is an exhaustion feature making the lymphocyte less active. This may explain the hypothesis as to why obese patients respond better to immunotherapy treatment. No differences were found in terms of Ob-R expression and pathological response by hormone receptor positivity or negativity (26).

## 5 BMI and prognosis in breast cancer survivors

Numerous studies and early systematic reviews/meta-analyses have examined the relationship between high BMI and early BC outcomes, and have shown that obesity is linked to lower survival (27–29). Chan et al. conducted a large meta-analysis to explore the magnitude of the associations in localized BC taking into consideration BMI in three treatment periods (before diagnosis, <12 months after diagnosis, and ≥12 months after BC diagnosis) and also in relation to menopausal status (30). A total of 210,000 BC survivors and 41,477 deaths were included in this analysis, and higher BMI was associated with poorer overall and BC survival in pre- and post-menopausal women, regardless of when BMI was determined, and being overweight was also related to a higher risk of mortality. Unfortunately, this study did not consider the risk according to BC subtypes. However, other studies which did investigate the relationship between high BMI, menopausal status, and BC survival/mortality according to type of BC yielded contradictory findings (31–33).

In this regard, Lohmann and co-workers analyzed the association between high BMI and BC outcome in relation to molecular subtypes in a literature-based meta-analysis (34). Overall, obesity was associated with modest, but statistically significant reductions in disease-free survival (DFS) and overall survival (OS) in all BC subtypes. Obesity was also associated with a statistically significant reduction in BC-specific survival in the hormone receptor-positive (HR+)/HER2-negative (HER2-) and TNBC populations. However, the situation regarding overweight is less clear to understand, since overweight was not associated with worse outcomes in the HER2+ or TNBC cohorts. Only 2 studies reported correlations between overweight and DFS in the HR+HER2-subgroup, so a meta-analysis was inappropriate. In a meta-analysis involving only 3 studies, a modest but statistically significant association of excess bodyweight with OS in the HR+HER2-subgroup was noted (HR= 1.14, 95% CI=1.07 to 1.22). Very few studies reported data with respect to menopausal status.

Elucidating the biological mechanisms by which excess adipose tissue leads to a worse prognosis in patients with early BC is the subject of ongoing research. The contributions of these mechanisms may differ across BC subtypes. Factors that may influence this negative prognosis include those related to excess adipose tissue per se, such as the increased estrogen production by the elevated numbers of adipocytes; this would be more relevant in HR+ tumors. Furthermore, comorbid diseases associated with obesity such as metabolic syndrome, diabetes, and cardiovascular disorders will likely have a negative impact on OS. In addition, people with obesity also tend to have larger tumors and increased lymph node involvement, factors associated with a poorer survival (35, 36).

Another possible explanation for worse BC outcomes in obese individuals is the potential for underdosing of chemotherapy if the dosage is capped rather than administered according to a bodyweight-based dosing regimen. Finally, as discussed earlier, interactions that adipose tissue may have with the cells that form the cellular microenvironment, especially those of the immune system, is another mechanism that could affect the response to treatment and therefore disease prognosis.

In patients with metastatic BC, limited data are available regarding the impact of BMI on clinical outcomes. One of the largest studies to date comes from the French ESME data platform, which included 12,999 metastatic BC patients at the time of analysis. Overall, overweight or obesity had no impact on overall survival whereas underweight was an independent negative predictor for OS (37).

## 6 BMI and its association with the response to breast cancer treatments

Optimal therapy for BC should be individualized taking into consideration factors such as tumor subtype, cancer stage, and patient preferences. For non-metastatic BC, the main goals of therapy are surgical removal of the tumor and also the regional lymph nodes (if necessary) so as to prevent metastatic development. Postoperative radiation is also usually advocated. Systemic therapy is dependent upon BC subtype and can be preoperative (neoadjuvant), postoperative (adjuvant), or both. Generally, treatment consists of endocrine therapy for all HR+ tumors (with some patients requiring chemotherapy as well), HER2-directed antibody therapy plus chemotherapy for all HER2+ tumors (with hormonotherapy given in addition, if HR+), and chemotherapy alone for TNBC. For metastatic BC, therapeutic goals are prolonging life and symptom palliation.

BC is more prevalent in patients with obesity and it is more likely to be associated with aggressive/advanced tumors that respond less well to treatment. It presents some unique challenges and it is a poor prognostic factor resulting in more complications, increased toxicity, and worse outcomes including relapse/recurrence and death (38, 39).

### 6.1 BMI and its association with the response to chemotherapy

Neoadjuvant systemic treatment is a common therapeutic approach for downstaging tumors/nodes in the management of early BC, and it has the advantage that the effects of treatment can be monitored using biomarkers predictive of in vivo tumor sensitivity and pathological response (40). Furthermore, pCR is a well-recognized predictor of survival benefit in TNBC and HER2+ patients (41). The impact of BMI on pCR with neoadjuvant chemotherapy was assessed in a pooled analysis of four prospective clinical trials (2 in HER2+ subtypes, 1 in TNBC patients, and the final trial involving all tumor subtypes) and found that, although obese women showed lower pCR in estrogen receptor-positive (ER+)/HER2+ and higher complete responses in ER-/HER2+ cancers, overall, there was no major difference in pCR rates depending on BMI. These authors concluded that pCR in patients receiving optimal doses of neoadjuvant therapy is a function of the tumor per se, rather than patient biology (42). In contrast, an earlier retrospective study in 246 consecutive female patients diagnosed with Stage II or III locally advanced BC and scheduled for surgery demonstrated that excess weight/obesity had a negative impact on pCR following neoadjuvant chemotherapy (43). The reasons for this poorer outcome in patients with high BMI need to be analyzed further in order to optimize the care of this group of patients.

As noted earlier, serum leptin levels are directly related to BMI, and people with obesity have higher leptin levels than lean people. Further, Ob-R positivity was also significantly higher in patients with a BMI >25 kg/m^2^ and a significantly greater percentage of patients with Ob-R positive tumors achieved pCR to neoadjuvant treatment compared to Ob-R negative patients (24). Currently, the reason why Ob-R overexpression predicts a higher pCR is not fully understood. However, we propose that it may stimulate signaling pathways associated with tumor proliferation, potentially increasing sensitivity to chemotherapy (24).

Despite the risks associated with adjuvant chemotherapy, full doses of cytotoxic therapy are associated with long-term survival benefits and are essential for greater overall clinical improvement and preventing recurrence in many patients with stage I-III BC (38, 39, 44). This is a potential issue for obese patients since appropriate dosing of cytotoxic therapy is challenging. This concern resulted in the American Society of Clinical Oncology (ASCO) publishing a guideline addressing dosing recommendations in obese adults (45) which has been updated in recent years to include findings for targeted and immune therapies (46). The ASCO guideline was based on a systematic literature review in adults with cancers such as breast, ovarian, colon, and lung cancer, and the expert panel noted that there were compelling data in patients with BC that reduced dose-intensity chemotherapy is associated with increased disease recurrence and mortality. They recommended that full weight–based chemotherapy doses (intravenous [IV] and oral) should be used in the treatment of obese patients with cancer, particularly when the goal of treatment is cure (45). However, even when the dosing of adjuvant chemotherapy is appropriate, patients with obesity have been found to have worse outcomes to BC treatment (38). Poorer responses to adjuvant chemotherapy in obese patients with BC was reported in the 30-year follow-up of the Danish Breast Cancer Cooperative Group (n=53,816). It was found that obesity was associated with an increased risk for developing distant metastases and dying from BC. Furthermore, the benefits of adjuvant chemotherapy were significantly less in obese patients, even among women who received appropriate doses of chemotherapy, and the findings were independent of tumor size, nodal status, and known prognostic factors (47).

### 6.2 BMI and its association with the response to endocrine therapy

Collectively ER+ and progesterone receptor-positive (PR+) tumors are commonly classified as HR+ BC. In HR+ disease, estrogen-activated estrogen receptors (ERs) initiate gene transcriptional programming that supports the aberrant proliferation of HR+ cancer cells that drive tumor formation and disease progression (48). It follows that the cornerstone of treatment of HR+ BC will focus on estrogen/ER signaling with anti-estrogen therapy or endocrine therapy (44). Indeed, endocrine intervention in hormone-sensitive BC remains one of the most important options in all settings of early and metastatic BC.

Endocrine therapy, which is designed to inhibit estrogen-promoted tumor growth, includes ER antagonists (e.g., tamoxifen) and aromatase inhibitors (e.g., anastrozole, exemestane and letrozole). Aromatase inhibitors reduce estrogen levels by inhibiting the conversion of androgens to estrogen and are only effective in postmenopausal women (44). In premenopausal women they should be used in combination with ovarian suppression. Aromatase levels have been shown to be elevated in obese patients and this could potentially reduce the effectiveness of aromatase inhibitors due to incomplete aromatase blockage. However, data from different studies that analyzed the increase of risk on disease recurrence in obese patients and their potential need for higher doses of aromatase inhibitors have been inconclusive (49).

To confirm whether aromatase inhibitors are less effective in obese patients, retrospective analyses were conducted in two randomized phase III studies, the ATAC study and BIG1-98 with conflicting results (50, 51). In a recent analysis of data from the Danish Breast Cancer Group involving more than 13,000 postmenopausal HR+ BC patients who received adjuvant aromatase inhibitor therapy, there was an association between obesity and risk of BC recurrence (sub-distribution hazard ratio, 1.44 [95% CI, 1.17-1.77]) compared with healthy weight patients (52). Overweight patients had a non-statistically significant greater risk of recurrence compared with healthy weight individuals (adjusted HR, 1.10 [95% CI, 0.97-1.24]). Follow-up for BC recurrence (contralateral BC, new malignant neoplasm, death, emigration, or end of clinical follow-up at 10 years) or end-date (September 25, 2018) began 6 months after surgery and continued until the first event of recurrence. Patients with any of these events were censored at the time the event occurred (52).

The biological explanation of this data is supported by pre-clinical evidence of incomplete suppression of estrogen levels using aromatase inhibitors in obese patients. These results raise the question of whether these patients would derive more benefit from tamoxifen or newer selective ER modulators (52). Several small, short-term studies have examined serum estrogen levels before and after treatment with aromatase inhibitors and confirm that patients with obesity have higher estradiol levels compared to patients without obesity (53, 54).

**Figure 1:**
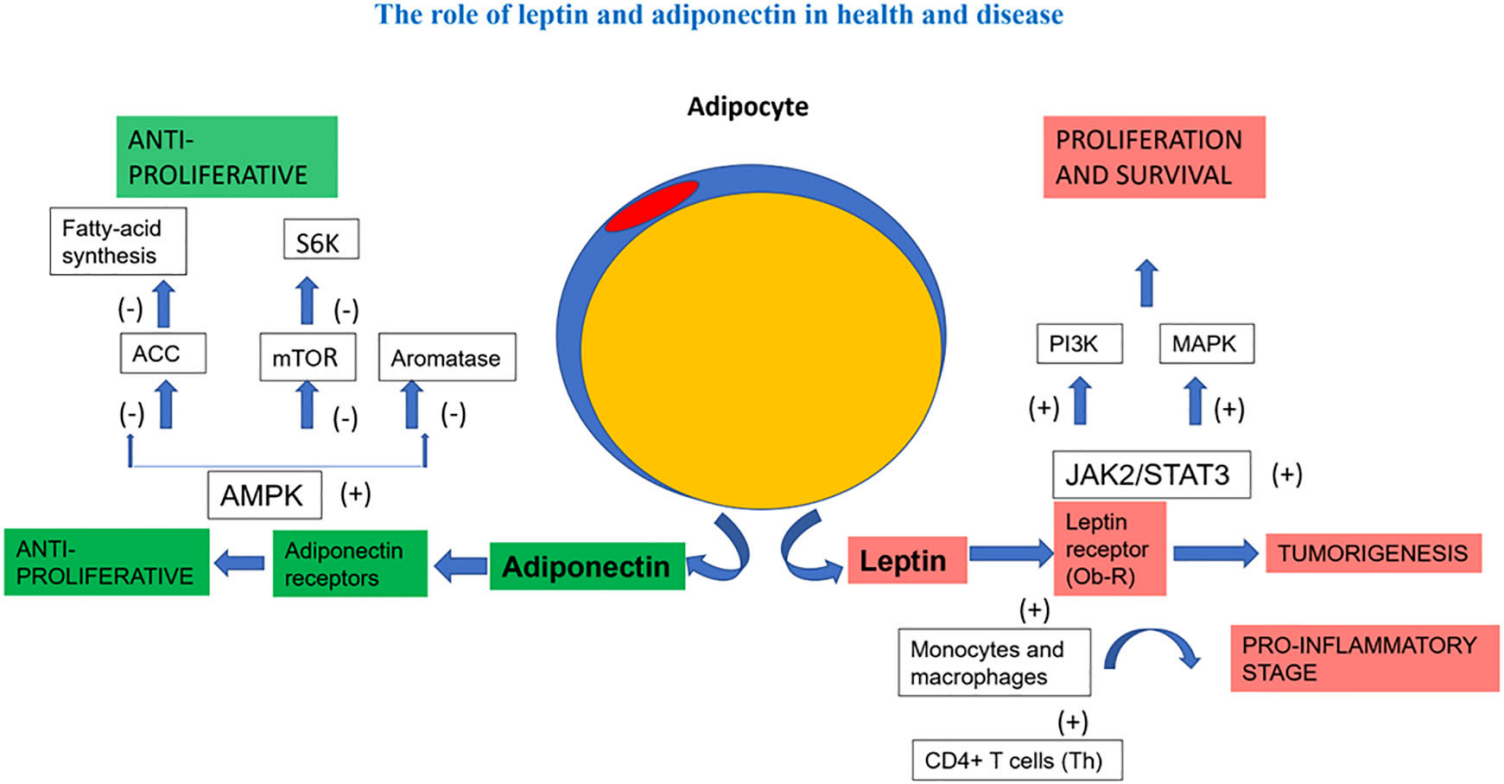
The role of leptin and adiponectin in health and disease. In a simplified manner, the figure demonstrates the antagonistic effect of adiponectin and leptin on inflammation and the different signaling pathways related to proliferation and survival. ACC, Acetyl-CoA carboxylase; AMP, activated protein kinase; JAK2/STAT3, Janus kinase2 / signal transducer and activator of transcription3 signalling pathway; MAPK, mitogen-activated protein kinase; mTOR, mammalian target of rapamycin; PI3K, phosphatidylinositol3-kinase; S6K, ribosomal protein 86 kinase.

Obesity can affect a number of other biochemical pathways which have the potential to reduce the effectiveness of endocrine therapy (38). These include: a) dysregulation of adipocytes which secrete adipokines (e.g., leptin), metabolites (such as cholesterol and free fatty acids), and cytokines (such as TNFα and interleukins) (**Figure 1**). The culmination of these effects is possible increased resistance to endocrine therapy through activation of various signaling pathways and regulating apoptosis-related genes. Certain adipokines and cytokines can also modulate estrogen synthesis by upregulating aromatase gene activity and have been found to diminish the effects of endocrine therapy *in vitro* (55, 56); b) patients with obesity tend to have higher insulin levels, leading to increased IGF-1 in BC cells. This can activate signaling pathways leading to endocrine therapy resistance (57); c) obesity is also associated with the overproduction of reactive oxygen species and proinflammatory mediators and these are known to further promote tumor progression (56, 58).

### 6.3 BMI impact on the response to targeted therapies

Recent advancements in targeted therapy, aimed at inhibiting tumor development, proliferation/progression, migration, and/or survival, have resulted in more specific and effective therapeutic options for managing certain BC subtypes. The most effective treatments for BC are those that target ER and HER2 receptors, but there is a rapidly growing body of evidence for other forms of targeted therapy including inhibitors of cyclin-dependent kinase (CDK) 4/6, poly (ADP-ribose) polymerase (PARP), Ak strain transforming (AKT), angiogenesis, and fibroblast growth factor receptors (FGFR) (59). Some targeted therapies, alone or in combination, have been approved by the FDA for the treatment of different BC subtypes, and many are also being investigated in clinical trials. However, drug resistance is a major challenge for these newer agents. Developments in this field are advancing quickly, but for some forms of targeted therapy, assessment of the impact of BMI on clinical outcomes and toxicity are lacking. For example, there is little or no information available for PARP inhibitors (e.g., olaparib and talazoparib), FGFR/tyrosine kinase/angiogenesis inhibitors (e.g., bevacizumab, lapatinib, neratinib, and tucatinib), and antibody-drug conjugates (e.g., fam-trastuzumab-deruxtecan and trastuzumab emtansine) to date (38, 59). Targeted therapies with available evidence are discussed below.

#### 6.3.1 HER2-targeted therapy

Trastuzumab, a monoclonal antibody that binds to HER2 receptors, was the first HER2-targeted therapy for BC and it has had a major impact in the treatment of HER2+ metastatic BC. In combination with chemotherapy, trastuzumab has increased clinical response rates and extended patient survival compared with chemotherapy alone in patients with HER2+ advanced BC (60). Trastuzumab also has therapeutic activity as monotherapy in the management of HER2-overexpressed or HER2-amplified metastatic BC. In the N9831 trial that compared chemotherapy with or without IV adjuvant trastuzumab in 3017 women with HER2+, early-stage BC, patients with high BMI had decreased survival compared to normal weight individuals (61). However, after stratification according to treatment administered, no differences in survival among the obese, overweight, and normal weight groups was observed. Despite the fact that the study was insufficiently powered to detect a statistical significance, it does indicate that IV adjuvant trastuzumab improves clinical outcomes regardless of BMI. In patients with HER2+ BC, (neo)adjuvant trastuzumab 600 mg subcutaneous (SC) every 3 weeks was non-inferior to 8 mg/kg IV loading dose followed by 6 mg/kg maintenance dose once every 3 weeks (62). However, PK models found that the first cycle of treatment with SC trastuzumab did not achieve target plasma concentrations in patients with an elevated BMI suggesting that a weight-adjusted IV dosage may be required for the first cycle (63, 64).

There are few studies investigating the impact of BMI on the efficacy and toxicity of newer HER2+ inhibitors. Oral HER2-targeted tyrosine kinase inhibitors such as lapatinib, neratinib, and tucatinib, as well as the mAb pertuzumab are all administered on a fixed-dose basis (38). In this regard the large, randomized phase III study NeoALTTO provided some interesting data in HER2+ early BC patients when evaluating the impact of BMI on pCR following treatment with lapatinib, trastuzumab, and their combination plus paclitaxel. In this study, BMI was associated with decreased pCR rates in HR+, but not in HR- cases. However, this result should be viewed in the context of the anthracycline-free chemotherapeutic regimen administered in this study (65). In a recent study, Chen et al. analyzed retrospective data from 491 HER2+ BC patients receiving neoadjuvant treatment and found that overweight/obesity was associated with a lower pCR. In addition, a meta-analysis of published literature was conducted in the same analysis, and it further confirmed the negative effect of BMI on pCR (66).

In contrast, the antibody-drug conjugates (ADC), trastuzumab emtansine and trastuzumab deruxtecan, are administered according to bodyweight, given that population pharmacokinetic studies have shown that bodyweight impacts both the volume of distribution and the clearance of these agents (67, 68). To date, there are no data regarding the association between BMI and ADC efficacy.

#### 6.3.2 Sacituzumab-Govitecan

Sacituzumab-Govitecan (SG) is a Trop-2–directed ADC approved in multiple countries for the treatment of relapsed or refractory metastatic TNBC. In the phase 3 ASCENT study (n=529), significantly longer progression-free survival (PFS) and OS were observed with SG vs chemotherapy treatment of physician’s choice (TPC) (69). One of the most interesting mechanisms of action of this drug, apart from the binding to its receptor and drug internalization, is the by-stander mechanism occurring in the tumor microenvironment. Garcia-Estevez et al, evaluated the association between BMI and efficacy in the phase 3 ASCENT trial to determine if the efficacy of SG might be lower in patients with obesity, as excess adipose tissue might interact with the by-stander effect. SG demonstrated improved efficacy vs TPC and had a manageable safety profile in all evaluated BMI subgroups. Although a larger proportion of patients with obesity (41%) had SG dose reduction, this did not translate into a decrease in efficacy (70).

#### 6.3.3 CDK 4/6 inhibitors

The current standard of care for many patients with ER+ metastatic BC consists of a CDK4/6 inhibitor combined with endocrine therapy (71). Three CDK4/6 inhibitors are currently available (abemaciclib, palbociclib, and ribociclib) which are administered in fixed-doses based on population pharmacokinetic studies, which showed no clinically significant effects of bodyweight on drug exposure (38).

Preclinical data suggest that CDK4 and CDK6 affect cell metabolism and the control of important metabolic processes such as adipogenesis and lipid synthesis, glucose regulation, and mitochondrial function (72). Based on these findings, it might be hypothesized that overweight and obese patients may have different efficacy and safety outcomes compared with patients with a normal BMI when treated with CDK4/6 inhibitors and endocrine therapy. Indeed, in a pooled analysis of patient data from the MONARCH 2 and 3 clinical trials, adding abemaciclib to endocrine therapy prolonged PFS regardless of BMI, showing that patients who are overweight/obese also benefit from this form of treatment (71). In the neoadjuvant setting, the combination of abemaciclib and endocrine therapy also seemed to be active regardless of BMI (71). In the adjuvant PALLAS trial evaluating endocrine therapy with or without palbociclib, BMI did not affect efficacy in terms of invasive DFS; however, patients with high BMI presented with a significant decrease in neutropenia and this translated into a significant decrease in treatment discontinuation rate (adjusted hazard ratio [HR] for 10-unit change, 0.75; 95% CI, 0.67 to 0.83) (73).

Other studies showing no statistically significant impact of bodyweight on clinical outcomes included adjuvant use of palbociclib plus endocrine therapy in early-stage BC; palbociclib or ribociclib (both with endocrine therapy) in metastatic disease; and abemaciclib, palbociclib, or ribociclib in metastatic HR+/HER2- BC (73–75).

#### 6.3.4 mTOR/P13K inhibitors

Activation of the phosphatidylinositol 3-kinase (PI3K)–AKT-mammalian target of rapamycin (mTOR) signaling pathway plays a key role in cellular growth, division, migration, and survival, including the development and progression of cancer cells. mTOR and PIK3 inhibitors prevent the downstream signaling required for cell cycle progression and proliferation. It is also associated with the development of resistance to endocrine therapy in BC patients (76). Limited data are available regarding the impact of obesity on the efficacy of mTOR and PIK3 inhibitors in BC patients. The mTOR inhibitor everolimus is prescribed as a fixed dose, based on findings from a study demonstrating that bodyweight does not affect the pharmacokinetic properties of the drug (77). Furthermore, in a real-world study, no correlation between baseline BMI and PFS was observed during treatment with everolimus plus exemestane in metastatic BC (78). The PIK3CA inhibitor alpelisib is also prescribed as a fixed dose, based on a study that showed no effect of bodyweight on its pharmacokinetic characteristics, and in one small trial (n=27) in which BMI had no impact on clinical response in patients treated with alpelisib for metastatic PIK3CA-mutated BC (79).

### 6.4 BMI impact on the response to immunotherapy

In recent times, BC treatment has focused on the presence or absence of molecular markers for estrogen, progesterone, and HER2 receptors. This ‘targeted focus’ has seen significant advances in clinical outcomes. However, in about 15–20% of BC cases the patient lacks all 3 molecular markers and are classified as triple-negative (44). Treatment of TNBC has long been challenging due to its aggressive nature and the lack of a specific therapeutic target. It is commonly diagnosed at a younger age compared with other BC subtypes and has a poor prognosis in case of metastatic relapse. A variety of treatment approaches are available for triple-negative tumors including chemotherapy, immunotherapy, radiotherapy, and surgery (80). Chemotherapy with drugs such as anthracyclines, cyclophosphamide, taxanes, carboplatin, and capecitabine has been the most widely used of these options, with neoadjuvant chemotherapy widely accepted as the standard-of-care for early TNBC to de-escalate the surgical procedure, preemptively predict tumor response and to enable adequate post-surgery treatment (81). However, relapse remains a clinical concern, and in the last couple of decades there have been multiple avenues of research to identify novel agents to improve the prognosis of patients with TNBC. At the forefront of this research effort is an evaluation of various immunotherapies to improve clinical outcomes and OS. The basis for this research is the finding that TNBC is potentially associated with a relatively high tumor mutational burden compared to other BC subtypes. This feature can serve as an immunogenic target for newer treatments such as immune checkpoint inhibitors (e.g., programmed cell death protein-1/programmed cell death ligand-1 [PD-1/PD-L1] blockers; cytotoxic T-lymphocyte-associated antigen-4 [CTLA-4] blockers (82).

Developments in this field are advancing quickly and, generally speaking, for most novel immunotherapies there is little information available regarding the impact of BMI on clinical outcomes and toxicity. Breast tumors are characterized by relatively low T-cell infiltration and apparent lack of response to immunotherapy, but the increased infiltration of regulatory T cells (Tregs), CD8+ T cells, and B cells in the TNBC tumor microenvironment has suggested that treatment with PD-L1 checkpoint inhibitors could induce a strong antitumor immune response in obese patients and a better prognosis (83, 84). Furthermore, in other cancers, adipose tissue excess in obesity has been linked to upregulation on T cells through immune checkpoints showing improved responses to immunotherapy agents (85). In this regard, our research on the HER2+ subtype may shed some light on why obese patients seem to benefit more from immunotherapy (26).

The impact of BMI on immunotherapy is poorly reported, although a systematic review of 18 studies (17 retrospective and 1 pooled analysis) involving patients with solid tumors reported mixed results in terms of survival outcomes and immune-related adverse effects after treatment with immunotherapy (86). Another systematic review of 13 retrospective studies which included patients with solid tumors treated with immune checkpoint inhibitors found a positive association between high BMI and improved survival (87). Well-designed studies evaluating the impact of BMI with targeted immunotherapies for BC are lacking.

## 7 Discussion

As noted earlier in the review, obesity has a marked impact on health and, in women, it is associated with an increased risk for many chronic conditions such as diabetes and heart disease. It also results in an increased prevalence of malignancies including BC. Although at present, the most practical and easiest way to measure excess adipose tissue is by BMI, the limitations of this tool are important because it does not measure differences in the quality of adipose tissue nor its distribution in the body. Future research should rely less on BMI and focus more on waist-to-hip ratio, body roundness index, and body composition findings gained from CT scans or other methods. With regard to BC, both genetic factors and an interaction involving the TME and tumor cells have been shown to be important. The microenvironment comprises a number of cell types including adipocytes which are known to be very active and responsible for the release of a variety of adipokines. The high proportion of adipose tissue in mammary glands constitutes a microenvironment that enables the immune system to remain responsive. Expression of Ob-R by T and B cells is of interest since it points to the potential involvement of leptin in immune cell activation and signal transduction. Indeed, obesity is associated with hyperleptinemia which is an established driver of metastasis in BC and has also been shown to promote angiogenesis through up-regulation of estrogen signaling (88). Further analysis of the processes involved could reveal new effects of leptin on as yet unexplored immune cell functions.

In an obese environment, the relationship between estrogens and adipokines, and the activation of specific signaling pathways, clearly differs between menopausal stages. Particularly interesting is the inverse relationship between obesity and BC risk during premenopause; however, no clinical strategy could contemplate weight-gain as a therapeutic approach in these circumstances for obvious reasons. Ultimately, gaining a deeper understanding of the mechanistic link between adipogenicity and tumorigenicity in breast tissues, as well as how obesity and adipose tissue inflammation interact with female sex hormones, may also prove to be an important area for bench research. Such investigations may allow us to identify more effective biomarkers than those currently available. Importantly, this will aid in the development of new therapeutic strategies aimed at reducing BC risk and enhancing patient outcomes in individuals who are obese. The ultimate aim of this research effort would be to develop a more personalized approach to treatment of all patients with BC.

Obesity in BC patients presents some unique treatment challenges since it is a poor prognostic factor resulting in more complications, increased toxicity and worse outcomes, including relapse/recurrence and death. Optimal therapy for BC should be individualized taking into consideration factors such as tumor subtype, cancer stage and patient preferences. LeVee & Mortimer suggest that the clinical proposals outlined in **Table 1** for the management of systemic therapies in BC patients with obesity should be carefully considered, and clinical trials focusing on the treatment and outcomes of patients with obesity and all stages of BC are needed to better inform future treatment guidelines (24, 38, 42, 43, 46, 47, 53–58, 65, 71, 73, 89–104).

**Table 1:** Concerns and considerations regarding systemic therapy in patients with breast cancer and obesity [adapted from LeVee & Mortimer (38) and published with permission of MDPI Basel, Switzerland in accordance with the STM Publishers Permission Guidelines February 19, 2024].

Systemic treatment	Mechanisms related to obesity	Dosing strategy	Treatment concerns in patients	Considerations for patients with obesity
Chemotherapy	BSA-based dosing strategies may not accurately estimate drug PK in patients with obesity (86) Chemotherapeutic agents can have different PK profiles in patients with obesity (e.g., lipophilic drugs with a high affinity for adipose tissue may have a higher volume of distribution in patients with obesity) (85, 86) Conflicting results regarding differences in pCR (41, 42) Worse outcomes to breast cancer treatment (37, 45)	BSA-based dosing	Risk of under- or over-dosing using BSA-based dosing formulas, which may lead to decreased efficacy and/or increased toxicity Leads to weight gain and cardiometabolic AEs	Use actual body weight in BSA-based dosing formulas Dosing adjustments for toxicity should be considered on a case-by-case basis, taking into account the specific antineoplastic agent, patient characteristics beyond BMI and comorbidities (44)
Endocrine therapy	Increased levels of estrogens due to aromatization of adipose tissue may lead to inadequate estrogen suppression with aromatase inhibitor therapy (51, 52) Unclear role of ovarian function suppression regarding BMI (89) Dysfunctional adipocytes release adipokines, metabolites, and cytokines, which induce endocrine resistance by activating various signal transduction pathways, modulating apoptosis-related genes, and upregulating aromatase activity (53, 54) Adipokines and cytokines have been found to directly diminish the efficacy of endocrine therapy *in vitro* (52). Increased insulin levels and IGF-1 activate the PI3K/AKT/mTOR and RAS/RAF/MAPK signaling pathway, leading to endocrine resistance (55, 90, 91) Chronic inflammation results in endocrine therapy resistance through the activation of proinflammatory molecules and reactive oxygen species (54, 56, 92, 93)	Fixed-dose	Increased endocrine-related toxicities and joint symptoms in patients with obesity Leads to cardiometabolic AEs and increased risk of VTE in patients with obesity	Choice of endocrine therapy should be made irrespective of BMI Consider comorbidities and cardiac risk factors when evaluating endocrine therapy choice and duration in the adjuvant setting
Trastuzumab	Further research is needed	Weight-based (IV) fixed-dose (SC)	The first SC dose may be suboptimal (61) Increased risk of cardiotoxicity and other AEs in patients with obesity	Consider a loading dose with SC or IV first loading dose administration for patients who are overweight and those with obesity
Pertuzumab	Further research is needed	Fixed-dose	NA	Treat irrespective of BMI
Antibody drug conjugates (T-DM1, fam-trastuzumab deruxtecan)	Further research is needed	Weight-based	Increased toxicity with TDM1 in patients with obesity	Treat irrespective of BMI
Tyrosine kinase inhibitors (lapatinib, neratinib, tucatinib)	BMI was associated with decreased pCR rates in HR-positive, but not in HR-negative cases (63)	Fixed-dose	NA	Treat irrespective of BMI
CDK 4/6 inhibitors (palbociclib, ribociclib, abemaciclib)	CDK4 and CDK6 help regulate cellular metabolism, including lipid synthesis, oxidative pathways, insulin signaling, glucose regulation, and mitochondrial function (70, 94–96) Overweight and obese patients had lower rates of neutropenia and discontinuation (70)	Fixed-dose	NA	Treat irrespective of BMI
mTOR and PI3K inhibitor (everolimus and alpelisib, respectively)	Activation of the PIK3–mTOR pathway results in insulin resistance and altered glucose metabolism (97, 98)	Fixed-dose	Associated with dyslipidemia, hyperglycemia (primarily alpelisib) (99)	Obtain serial fasting blood sugars and lipid panels For alpelisib only: • Optimize blood glucoses prior to initiation of alpelisib • Counsel on healthy lifestyle behaviors and symptoms of hyperglycemia • Closely monitor for signs and symptoms of hyperglycemia to allow for the early detection and management of hyperglycemia-related complications
PARP inhibitors (olaparib, talazoparib)	PARP enzymes help regulate metabolic pathways, including carbohydrate and lipid metabolism and adipocyte differentiation (100)	Fixed-dose	NA	Treat irrespective of BMI
Trop-2-directed antibody-drug conjugate (sacituzumab govitecan-hziy)	Further research is needed	Weight-based	NA	Treat irrespective of BMI
Immunotherapy (pembrolizumab)	Excess adipose tissue results in immune system dysfunction (81) Patients with high BMI may have better outcomes, overall response rate and longer PFS (101)	Fixed-dose	NA	Treat irrespective of BMI

AE, adverse event; AKT, protein kinase B; BMI, body mass index; BSA, body surface area; CDK, cyclin-dependent kinase; IGF-1, insulin-like growth factor-1; IV, intravenous; MAPK, mitogen-activated protein kinase; mTOR, mammalian target of rapamycin; NA, not applicable; pCR, pathologic complete response; PI3K, phosphatidylinositol3-kinase; PARP, poly(ADP-ribose)polymerases; PFS, Progression-free survival; PK, pharmacokinetics; RAF, rapidly accelerated fibrosarcoma; RAS, rat sarcoma; SC, subcutaneous; T1DM, type-1 diabetes mellitus; VTE, venous thromboembolism.

Given the large numbers of women developing BC and the high mortality rates associated with the disease, there remain a number of unmet needs to try and improve clinical outcomes. From a clinical perspective, the foremost priority is the necessity for improved treatment options. In this respect, research is advancing rapidly with many targeted therapies for specific signaling pathways and immunotherapies being developed. Given the association between obesity, overweight, and BC, there may also be future potential for the use of metabolic-targeting drugs such as metformin and glucagon-like peptide-1 receptor agonists, although this remains an emerging area of research (105, 106). Currently, the limited data on the use of these agonists in BC patients receiving hormone therapy have demonstrated a lower efficacy in terms of median weight reduction achieved compared to patients without cancer and therefore without such hormone therapy (107).

In conclusion, the optimal use of these agents, alone or in combination, is an important avenue of future study. This will include a focus on dosage, which as noted in this review, is a key criterion to get right for many anticancer treatments.
